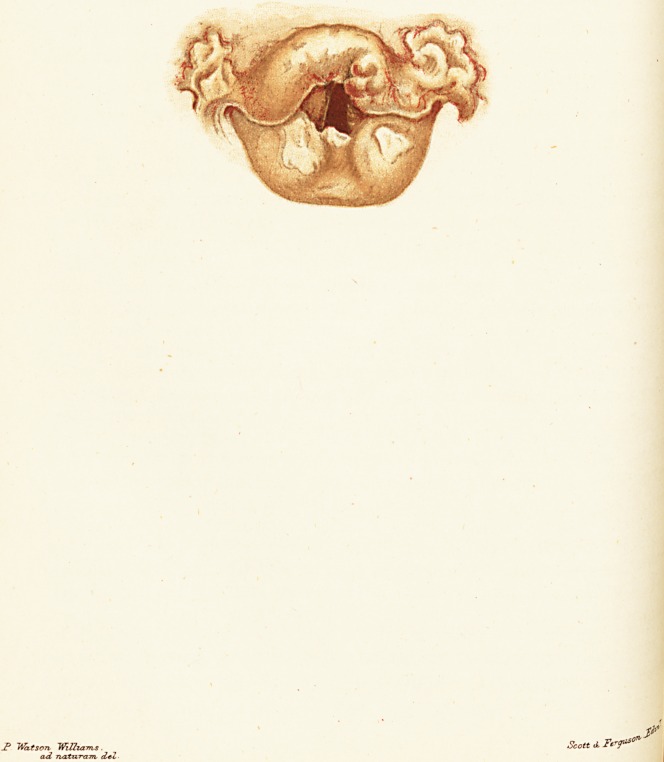# The Combination of Syphilis and Tuberculosis, Especially in Regard to Laryngeal Affections
^1^Read at the Bath and Bristol Branch of the British Medical Association, October, 1892.


**Published:** 1893-09

**Authors:** P. Watson Williams

**Affiliations:** Physician in charge of the Department for Diseases of the Throat, Bristol Royal Infirmary


### THE COMBINATION OF SYPHILIS AND TUBERCULOSIS, ESPECIALLY IN REGARD TO LARYNGEAL AFFECTIONS.[1]

BY

P. WATSON WILLIAMS, M.D. Lond.,

*Physician in charge of the Department for Diseases of the Throat, Bristol Royal Infirmary.*

---

"It appears to me, beyond a doubt, that no two (morbid) actions can take place in the same constitution, or in the same part, at one and the same time. . . . Yet as the venereal disease, when it attacks the skin, bears a resemblance to those symptoms which are vulgarly called scorbutic, they are often supposed to be mixed and to exist in the same part." Thus wrote the great John Hunter[1] in 1788, and from this quotation we may infer that the condition to which I wish to direct attention was the subject of opposing views a century ago.

[1] Read at the Bath and Bristol Branch of the British Medical Association, October, 1892. [2] *A Treatise on the Venereal Disease*, p. 2.

The question as to whether such a combination really exists had rather passed out of the active consideration of the profession, till it was again mooted by Ricord and by Verneuil at the International Medical Congress in 1881. But the experimental and clinical investigations of Professor Leloir, of Lille, brought before the Congress for the Study of Tuberculosis in Man and Animals at Paris in 1891, appear to leave absolutely no doubt that cases of a hybrid disease, which he terms scrofulo-tuberculo-syphilitic, actually do occur. It is still, however, commonly believed that while syphilis and tubercle may run a parallel course in the same individual, they do not amalgamate, but remain distinct.

Professor Leloir had a patient who presented ulcerations which showed certain special features in their character and progress. He found that they were made to heal partially under the influence of anti-syphilitic remedies; and, as he remarks, it is only reasonable to assume that the lesions were partly syphilitic. But arrived at this point, the process of cure was arrested. He then came to the conclusion that what remained uncured, not being truly syphilitic, was in fact tubercular; and to leave no doubt on the point, he had recourse to inoculations of guinea pigs with fragments from the ulcers, and these guinea pigs very shortly presented all the characteristic signs of tuberculosis, a uniform result in several animals thus inoculated. He concluded that this was direct evidence in support of his hypothesis that that which anti-syphilitic treatment had not made to disappear was in relation to scrofulo-tuberculosis.

In this way he demonstrated by experimental research, by histological and bacteriological examination, and also by clinical observation and therapeutical results that, in certain cases, certain adenopathies and ulcerations occurring in syphilitic subjects are at the same time tubercular and syphilitic, and constitute a true hybrid affection.

But if we admit that lupus is a form of tuberculosis of the skin, even more conclusive evidence of the existence of a hybrid affection was furnished by a case of mixed lupus and syphilis of the skin. The patient presented lupus tubercles of a peculiar character; they were about the size of half a hazel nut, hard and copper coloured. Histological examination of sections of portions removed showed the presence of giant cells and of tubercle bacilli in their neighbourhood. But the arterial coats were thickened, and the structure of the deposit was very suggestive of syphilis. Some of this morbid deposit disappeared completely under iodide of potassium and mercurial inunction, while the tissue that remained uncicatrised after syphilitic treatment was perfectly cured by scraping and lactic acid.

As Professor Leloir remarks, it is the first case of the hybrid lupus and syphilis which has been demonstrated and published.

There are good reasons to expect that such an association of tubercular and syphilitic disease should occur most frequently and typically in the fauces and larynx, and perhaps also in the nasal mucous membrane, since these regions may be regarded as the common seat of election for the manifestation of the grosser lesions, and particularly ulcers in syphilitic and phthisical patients. Yet very few such cases have been described, and in only a few works is the question considered. Probably no one doubts that syphilis and phthisis occurring in the same subject mutually exercise a marked influence on the evolution of each affection respectively; few of us would hesitate to say that this mutual interaction is ever otherwise than pernicious.

It is only comparatively recently that the possibility of the occurrence of the two affections simultaneously in the larynx has been at all generally accepted, and it is especially due to Professor Schnitzler, of Vienna, that this interesting combination has attracted our attention, although in 1876 Sir James Paget said, "I would not venture to call the disease that may occur in a scrofulous person, become syphilitic, a hybrid one: and yet, perhaps, the term is not altogether wrong; but, at least, I would call it a mixed disease, and hold that syphilis inserted in a scrofulous person will, in its tertiary period, produce . . . . signs in which the characters of scrofula and of syphilis are mingled, and which . . . . require that the treatment of scrofula should be combined with the treatment of syphilis, in order to produce a fully successful result."[1]

[1] *Trans. Pathological Society*, Vol. XXVII., p. 371.

The diagnosis of syphilis and tuberculosis seldom offers any difficulty to the laryngologist, for the practised eye can at once determine the true nature of the disease in the majority of cases; but, as Schnitzler recently remarked, "there are, however, cases which come under our notice that are not so easy of recognition. The differential diagnosis between the two morbid processes is often very difficult, if not impossible, and thereby affects our treatment of the case";[1] and he has described cases in which syphilis and tuberculosis are combined in the larynx, the one running parallel with the other, or where tubercular ulcers have been rapidly followed and even displaced or transformed into syphilitic ones, or *vice versâ* where the syphilitic has been changed into the tuberculous. "The two ulcers may commence simultaneously, extend and coalesce, and remain to the end two distinct ulcers."

As an example of such a hybrid affection occurring in the larynx, I give the following case in a young man, age 24:—

Two years before I saw him, he had consulted his medical attendant for cough, night sweats, and loss of flesh, and was told that his lungs had a tendency to be affected. He improved greatly under treatment. Eighteen months later, he was being treated for mucous tubercles about the anus. Six months after that, he noticed that his throat was a little sore on swallowing and he was hoarse in the evening, but there was no pain on talking, and he was again treated by anti-syphilitic remedies—at first with some improvement. When I saw him for the first time a month later, he complained of pain on swallowing. His voice was only a little hoarse, he was losing flesh rapidly, with night sweats, cough, and expectoration, which contained numerous tubercle bacilli. Temperature varied between 100° and 101°; pulse rate, 90. The apices of the lungs were affected, more especially the left, with dulness, prolonged expiration and increased vocal resonance, and occasional moist sounds. When he first came under my observation, the larynx showed a general acute inflammatory œdema without ulceration. But in spite of every endeavour to check the downward course, he rapidly lost flesh and the larynx became extensively ulcerated. His lungs became more extensively diseased, and he rapidly lost ground. Neither general nor local remedies to combat the tubercular affection, nor the administration of anti-syphilitic remedies, were of any avail, the combination of active syphilitic disease was beyond all power of control. Three months after

[1] *Med. Press and Circ.*, Jan. 14th, 1891.

*P. Watson Williams. ad naturam del.*

*Scott & Ferguson Edin*r

I first saw him, his larynx presented the appearance shown in the coloured plate. The epiglottis was thickened, infiltrated with tubercular deposit, and extensively ulcerated. But some of the ulcerations on its upper surface and extending to the lateral glosso-epiglottic folds were certainly highly suggestive of syphilitic disease. The condition of the arytenoid mucous membrane and the growths in the inter-arytenoid fold were typically tubercular in appearance. In other parts it would have been impossible to say definitely that they were syphilitic or tubercular in character.

This case, in its history, course, and in the laryngoscopic appearance, resembled very closely the first case recorded by Schnitzler in 1868, in which the *post-mortem* examination was made by Rokitansky. "It was interesting to see Rokitansky pointing here to a tubercular ulcer, there to a syphilitic, side by side; and sometimes one ulcer could be demonstrated to be composed of two different characters, which formerly might have been different, but now coalesced and forming a compound ulcer." Schnitzler states definitely that when this mixed condition occurs one cannot with any degree of certainty say whether it is syphilis or tuberculosis. "Of the correctness of this opinion, that it is difficult to separate the two morbid processes in diagnosis, I am more convinced every day; and after years of observation, I would say that it is possible for the two morbid processes to become so amalgamated that recognition is beyond our power. There are a great number of cases closely allied in appearance at one time, that can easily be recognised by some prominent symptom later on; where we can safely say: 'Here is a syphilitic case, there is a tubercular case;' but there still remains a class of cases where we cannot correctly apply this discriminating difference."[1] These remarks are in exact accord with my experience.

There is one point in connection with the combination of these diseases which I must just allude to; viz., the possibility that late syphilis may exercise a beneficial and not a pernicious influence on the course of phthisis. At the Congress at Paris to which I have already referred, Boulland, of Limoges, discussed the influence of kaolin dust in producing a form of true

[1] *Loc. cit.*

tubercular fibroid phthisis in the porcelain workers, and which sclerosis, or formation of fibrous tissue, seemed to exercise a most beneficial action in restraining the activity of the tubercular process, thus modifying its course and prolonging the life of the consumptive, if not actually producing a cure. Landouzy, however, considered that among other sclerogenous factors—factors tending to cause cicatrisation—we must include late syphilis. Thus in at least a dozen cases in which phthisis supervened on a syphilis of say twenty years standing, the course of the tubercular disease was peculiar and distinctive, clinically the affection being remarkably slow in development, apyretic, and with a tendency to remain localised, and pathologically belonging to the class of fibroid phthisis.

For my own part, I have always regarded syphilis, whether recent or old, as undoubtedly exercising a pernicious influence; yet, without adopting the opposite view, it is easy to recall many cases which lead one to think it possible that Landouzy's views may be justified.

The practical import and pathological interest attaching to the relatively rare cases of such hybrid combination in laryngeal affections are immense, when we realise the important bearing they have on the general question of combined syphilis and tuberculosis. In the larynx, we can watch the process during life; in the lung and other viscera, we can at best see the ultimate result on the *post-mortem* table, when generally there can be nothing sufficiently definite and striking in appearance to lead one to look for or suspect such a hybrid condition.

Gross syphilitic lesions are seldom seen on the *post-mortem* table; while tubercular deposits and destructive processes are so extremely common, that it would be very easy to overlook the syphilitic element in those comparatively few cases where this hybrid affection may exist. It is probable that they are more frequent than has hitherto been suspected, and perhaps if the existence of the hybrid affection to which I have drawn attention is kept before us we may find an explanation of some obscure and difficult cases in practice and obtain useful pathological hints.

## Figures and Tables

**Figure f1:**